# Preliminary investigation reveals novel pathological consequences of bluetongue virus-1 infection in the endocrine glands of pregnant Indian sheep

**DOI:** 10.1080/10495398.2023.2269428

**Published:** 2023-10-18

**Authors:** Rohit Singh, Karam Pal Singh, Rajendra Singh, Vidya Singh, Pawan Kumar, Rajat Varshney, Akanksha Yadav, Akash Mote, Mukesh Gangwar, N. Babu Prasath

**Affiliations:** a ICAR-Indian Veterinary Research Institute, Bareilly, India; b SOA Institute of Veterinary Science and Animal Husbandry, Bhubaneswar, India; c Department of Veterinary Microbiology, Faculty of Veterinary and Animal Sciences, I.Ag.Scs, RGSC, Banaras Hindu University, Mirzapur, India

**Keywords:** BTV-1, endocrine glands, Indian local pregnant sheep, histopathology, immunohistochemistry, real-time PCR

## Abstract

Bluetongue virus (BTV), a major peril to the sheep industry, infects a wide range of the cells in the infected animals including mononuclear, dendritic and epithelial cells. However, little is known about its tropism for the secretory epithelial cells of endocrine glands and the pathogenesis it induces. The aim of the study was to assess the BTV load, antigen distribution in the tissue of the pituitary, thyroid as well as adrenal glands and associated histopathological consequences. BTV antigens were localized using immunohistochemistry in the thyroid’s epithelial cells, zona fasciculata and zona reticularis cells and the anterior pituitary epithelial cells. The real-time PCR portrayed the high viral load in adrenals at 7^th^ days postinoculation (DPI) and in thyroid and pituitary glands at 15^th^ DPI. Serum examination revealed variation in the T-3 and T-4 of infected animals in comparison to the control group. Caspase-3 immunolocalization revealed BTV-1 induces apoptosis in the affected cells of endocrine gland of infected animals. Further, this study signifies the tropism of BTV in the novel sites (endocrine glands) of the host that might be one of the reasons for the poor performance of infected animals.

## Introduction

Bluetongue (BT), a noncontagious, Culicoides-transmitted viral disease of domestic and wild ruminants, poses enormous socio-economic losses worldwide. The clinical manifestations are frequent in sheep and other wild ruminants, while a subclinical form is seen in cattle, camels and goats. So far, 29 serotypes are known worldwide, of which 23 have been reported from India.^1^ The double-stranded, segmented RNA genome of bluetongue virus (BTV; Orbivirus genus; *Reoviridae* family) implicated in BT encodes seven structural (VP1–VP7) and five nonstructural (NS1, NS2, NS3/NS3A, NS4 and NS5) proteins.^2^^,^^3^ Its seg-2 and seg-6 determine the serotypes of the virus. Serotypes 1–6 and 10 are frequently associated with clinical disease with high pathogenic potential.^4^^,^^5^ Previous research data revealed that the seroprevalence of BTV-1 serotype in different geographic regions of India is quite high. Additionally, the potential of BTV-1 to induce transplacental transmission was relatively high in comparison to BTV-8 and pathological consequences induced by BTV-1 was relatively more severe.^6^^,^^7^

The BTV replicates both in Culicoides cells and in the vertebrate host cells. In vertebrate hosts, the virus replicates in leucocytes, lymphoid cells, endothelial cells and other epithelial tissues of the skin, lungs and other organ systems. The secretory epithelial cells of endocrine glands also support BTV replication, but its pathobiology is unclear. The BTV inflicts injuries to the endothelial cells of small blood vessels in various target tissues, causing cytolysis and the release of virus-induced vasoactive mediators. These mediators set the inflammatory process and account for extensive haemorrhages including at the base of the pulmonary artery, formation of ulcers in the digestive tract; necrosis of striated muscles (skeletal and heart); coronitis; oedema of the lungs, hypodermis and facia of various organs; effusions in the body cavities; age-dependent outcomes in foetuses—abortions/stillbirths; cavitating lesions in the central nervous system; viraemic newborn, weak born young ones; reduced fertility rate; meat and fleece losses.^8^ Besides these direct economic losses, indirect losses also result mainly due to the trade restrictions on animals/animal products; and expenditure for pest control, diagnosis, vaccination as well as treatment of clinically ill animals.^9^^,^^10^ In survivors, squeal may include a loss of body condition, deformities of hooves and abnormal wool growth as well as poor-quality semen.^11^

BTV may induce alteration in the host immune response via activation of the hypothalamic–pituitary–adrenal axis/stress system, which in turn may cause damage of endocrine gland. Further, viral-mediated modulation of the host endocrine signalling system had also been documented during viral infection. Mumps, Hantana, Herpes simplex virus-1 and -2 and HIV-1 implicated in the direct destruction of the affected endocrine glands.^12–15^ The autoimmune-mediated destruction of the endocrine glands has been reported in Rubella, Hepatitis-C, Influenza virus, Coxsackie B virus, enterovirus and Epstein Bar Virus infection.^16–18^ Moreover, viral proteins that alter/affect/inhibit the endocrine signalling of the host has also been described.^19–21^

Unlike in other viruses, the effect of the BTV on endocrine glands is obscure; therefore, the effect of the BTV-1 infection in the secretory epithelial cells of endocrine glands viz. pituitary, thyroid and adrenal glands with respect to their morphological changes, immunohistochemical antigen localization and virus replication, if any, in experimentally infected Indian pregnant sheep has been delineated in this study.

## Materials and methods

### Virus

The BTV-1 (BTV-1 SKN-10/India/2007) procured from the Virus Repository of ICAR-Indian Veterinary Research Institute, India was originated from the spleen of a goat aborted foetus at Livestock Farm, Surdarkrushi Nagar, Gujarat.^22^ Baby hamster kidney (BHK)-21 (vertebrate cell line) passaged BTV-1 inoculum was further cultured in KC cell lines (insect cell line) for a week and then harvested for experimental use in sheep. Viral titration was performed in the BHK-21 cells line by using Reed and Muench method for endpoint titration assay to determine the tissue culture infective dose (TCID_50_).

### Animals

Total of 15 ewes procured from the local animal vendor was screened for BTV antibodies in serum by using c-ELISA (VMRD) and absence of BTV viral RNA in blood by using RT-PCR before inoculation of virus (BTV/SKN-10). Out of 15 BTV-free ewes, 10 were used for experimental inoculation of BTV-1, while the remaining 5 were inoculated with the cell culture media. Prior to the experiment, all the animals were dewormed and housed separately (Control group and infected group) in a well-drained, well-ventilated and insect-proof facility of the Division. The sheep were provided with ad-libitum water and standard feed throughout the experimental period. The sheep were kept for acclimatization for 2 weeks and the behavioural responses were monitored. The present trials were conducted after obtaining approval of the CPCSEA (No. 25/21/2017) EC [No. F.26-1/2015-16/JD (R)]. The animals related experimental procedures have strictly adhered to the Guidelines of the Institutional Animal Ethics Committee and carried out under the guidance of the institutional animal care committee.

### Virus inoculation

**Figure 1.**
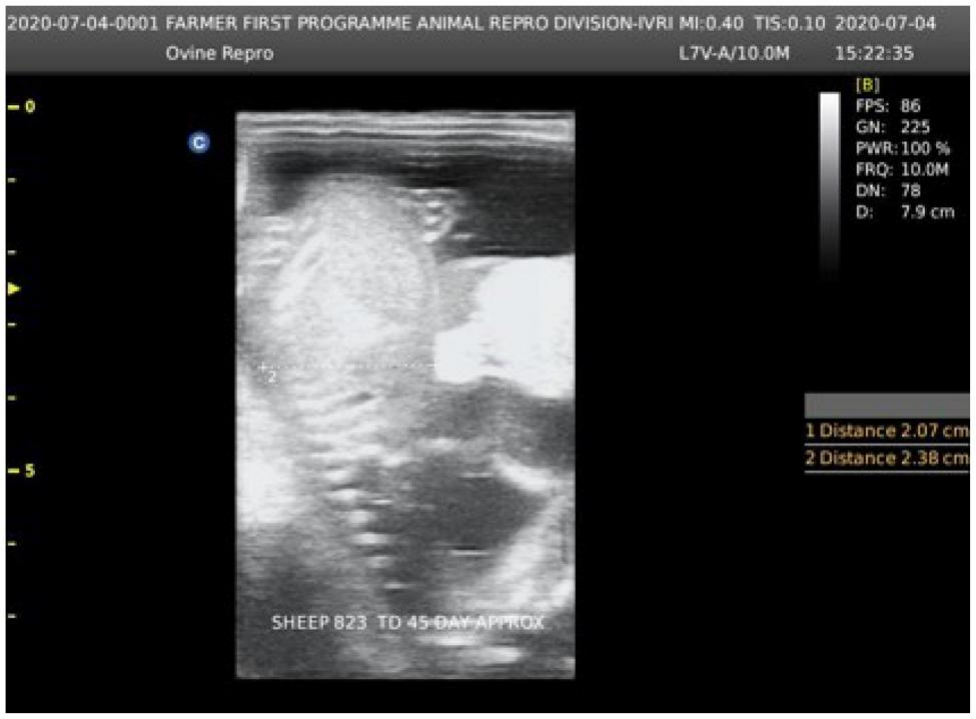
Fetal age determination by using (C3-A) real-time ultrasound equipment (Aeroscan-CD-5 Konica Minolta, India) having a convex transducer (C3-A).

The mated ewes were tested for pregnancy via real-time ultrasound equipment (Aeroscan-CD-5 Konica Minolta, India) having a convex transducer (C3-A; Fig. 1). Out of 15 pregnant sheep, 10 ewes at the 60^th^ day of gestation were administrated with BTV-1 virus @ 6 ml (5.5 log10 TCID_50_/ml) through an intradermal route using a 26-gauge needle at various sites on both right and left sides of prescapular/neck region, followed by 4 ml BTV-1 inoculums (5.5 log10 TCID_50_/ml) to each ewe intravenously as an infected group. The remaining five pregnant ewes (control group) at the 60^th^ day of gestation were injected intradermally mock cell culture fluid @ 6 ml/sheep at numerous sites in the prescapular/neck region, followed by 4 ml mock cell culture fluid intravenously.

### Blood samples’ collection and hormone estimation

Blood samples (∼2ml) were collected from the control and the experimental BTV-infected animals at specified time intervals by jugular vein venepuncture using BD Vacutainer (EDTA coated) for the estimation of hormone concentration. The blood samples were subjected to centrifugation at 1500 × *g* for 15 min in a hanging rotary centrifuge and sera were pipetted out in polystyrene tubes. Serum samples were stored at −20 °C until further use.

Progesterone, cortisol, and T-3 and T-4 concentrations in the serums were estimated by the ELISA (Biogenix Inc. Pvt. Ltd.) as per the instruction of the manufacturer. The OD value of each sample was calculated by using the ELISA reader (Thermo Scientific, USA) at the prescribed wavelength as recommended with each kit. The OD value was converted in the hormone concentration using the prescribed methods provided with the kit. Viral infection in the animals was confirmed by the real-time PCR at 0^th^ days postinoculation (DPI).

### Sacrifice of animals

BTV inoculated ewes (2 animals/interval) and control ewes (1 animal/interval) were sacrificed at the 7^th^, 15^th^, 30^th^, 45^th^ and 60^th^ DPI in the postmortem facility of the Division of Pathology, by injecting an overdose of the thiopental sodium intravenously. The study is conducted for preliminary investigation of the hypothesis. The small sample size was prompted by the first examination of the hypothesis. For this limited number of animals as per the availability of funds was considered for the study.

The pituitary, thyroid and adrenal samples were collected immediately in 10% neutral buffered formalin (NBF) from scarified ewes. The NBF fixed tissue samples were further processed for routine paraffin wax embedding technique, and 4–5 µm thick sections were prepared using a microtome. The tissue sections on the glass slides were stained with the routine haematoxylin–eosin (HE) method as per the standard staining procedure. For the viral quantification, thin-tissue sections were collected in the RNA Later and stored at −20 °C till further use.

### Genomic detection of BTV

Total RNA was extracted from 300 μl tissue suspension using Trizol^®^ reagent (Life Technologies, USA) as per the manufacturer’s instruction. The quantity and purity of RNAs were determined by measuring *A*_260_/_280_ using Nanodrop ND-1000 Spectrophotometer (Nanotechnologies, USA). The *A*_260/280_ values of all extracted RNAs were in the range of 1.8–2.0. These extracted RNAs were subjected to the genomic detection of BTV using Taqman-based chemistry through one-step RT-PCR (Qiagen, USA), using BTV-NS-3 gene primers and probes for seg-10/NS3 (F:5′-TGGAYAAAGCATGTCAAA-3′; R: 5′-ACRTCATCACGAAACGCTTC-3′ and 6-FAM-ARGCTGCATTCGCATCGTACGC-Tamra-Q.^23^ Real-time PCR amplified an amplicon of 78 bp in size. The reaction mix of 20 μl was prepared by mixing 5x reaction mix (2.0 μl), dNTP (0.4 μl), 0.5 μl of each primer and probe (10 pM), enzyme mix (0.4 μl), template RNA (1.0 μl) and RNase-free water (4.7 μl). The amplification was carried out in a AriaMx Real-Time PCR System (Agilent, USA). The thermal conditions were: reverse transcription at 50 °C for 30 min, followed by initial PCR activation at 95 °C for 15 min, template denaturation at 94 °C for 30 sec, primer annealing at 56 °C for 30 sec and extension at 72 °C for 30 sec, followed by a final extension at 72 °C for 10 min. During each reaction, no template (negative control) and controls with positive BTV RNA (positive control) were always used in duplicate to rule out contamination in reagents. Further, 5 μl of the amplicons were electrophoresed in agarose gel to confirm the specific product and to document the same using a gel documentation system (Azure Biosystem-C 300, USA). A standard curve was used for the calculation of the copy number of viral genome in tissue. The PCR efficiency (*E* = 100 × 10(−1/slope) was determined by evaluating the slope of the linear regression connecting the Cq value with the logarithm of the concentration (copies per reaction). The efficiency of real-time PCR was nearly 100%.

### Immunohistochemistry

The BTV antigens have been portrayed in endocrine glands via the indirect immune peroxidase technique as described earlier.^24^ Hyperimmune sera were raised in rabbit by immunizing the BTV-1 core antigen at the Mukteshwar campus of the Indian Veterinary Research Institute (IVRI). Primary antibodies obtained from immunized rabbit were BTV-1 specific and were subsequently used in immunohistochemistry (IHC). The endocrine gland samples from BTV experimentally infected animals and negative control animals were embedded in paraffin and paraffin-embedded blocks were sliced into thin sections (4–5 µm). The tissue section from the uninfected animals serves as a negative control for the IHC. The thin-tissue sections (4–5 µm) on 3-aminopropyltriethoxysilane (Sigma Aldrich, USA) coated slides were deparaffinized and rehydrated through descending grades of alcohol viz., 100%, 90%, 80%, 70%, 60% and 50% and finally kept in the distilled water. For antigen retrieval, the tissue sections were incubated with the proteinase-K (Sigma, USA) for 1 h at 37 °C. Further, tissue sections were washed using 1× PBS (pH 7.4) thrice for 5 min each. Tissue endogenous peroxidase activity was inhibited with freshly prepared 3% hydrogen peroxide (H_2_O_2_) in methanol for 30 min in a dark chamber, followed by 3× washing using 1× PBS (pH 7.4).

The tissue sections were further incubated with 10% bovine serum albumin (BSA) for 1 h at RT followed by washing in 1× PBS (thrice). Further, 150 μl of rabbit origin BTV specific serum was added to the sections at 1:20 dilution in 1% BSA and incubated in a humidified chamber at 4 °C for overnight. Sections were thoroughly washed with TBS thrice for 5 min each to remove the unbound antibody and incubated with biotinylated goat anti-rabbit IgG peroxidase conjugate (Sigma, USA) for 1 h at 37 °C, followed by washing thrice as above. The positive immunoreaction was identified by the development of brown colour with freshly prepared 3,3′-diaminobenzidine (DAB, Vector Laboratories, Burlingame, California). The sections were washed and counterstained with Mayer’s haematoxylin (Sigma Aldrich, USA) for 10–20 sec, followed by dehydration in the ascending grades of alcohol and then mounted with DPX mountant (Sigma, USA). Positive signals (brown colour) were recorded under the light microscope.

### Immunolocalization of the caspase-3

Like the BTV-1 IHC detection, the IHC staining was carried out for the antigenic demonstration of caspase-3 (Sigma, USA) in the tissue sections of the thyroid, adrenal and pituitary glands.

### Statistical analysis

Statistical analyses of data were performed using GraphPad Prism 6.0 software. Data were presented in the form of mean ± SEM. The statistical significance difference between the mean of the infected and control groups was verified by one-tailed, paired *T*-tests. *P* value <0.05 was considered as a statistical significance.

## Results

### Gross changes in endocrine glands of infected group

The noteworthy gross changes in pituitary of experimentally infected ewes were not observed at 7^th^, 14^th^, 30^th^, 45^th^ and 60^th^ DPI; however, mild congestion was observed in the pituitary stalk (infundibulum) at 14^th^ DPI. The gross changes in thyroid glands were also not remarkable. At the 7^th^ DPI, adrenal glands showed slight vascular congestion in the adrenal cortex. At 14^th^ DPI, the vascular congestion became intensified with thickened and oedematous cortex, while the contralateral adrenal cortex, in addition, showed haemorrhages and congestion of the medulla. At subsequent intervals, the gross changes in adrenal glands were not remarkable, when compared with the control.

### Histopathological changes in endocrine glands

#### Pituitary gland (hypophysis)

**Figure 2.**
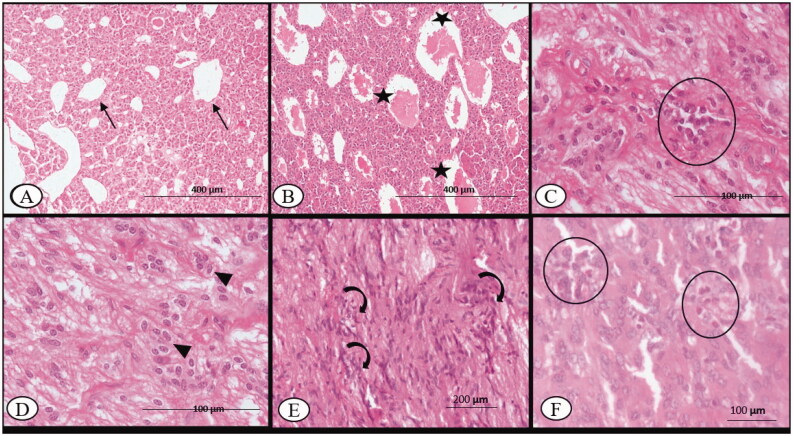
Bluetongue virus serotype-1 (BTV-1), 6 ml (5.5 log10 TCID_50_/ml) by I/D and IV routes, pregnant sheep at 60^th^ gestation day. Pituitary sections: (A) at 7^th^ DPI, showing normal capillary plexus (arrow) at C-7 DPI in the infected group, ×100; (B) at 7^th^ DPI, showing dilated venous capillary (asterisk) plexus with blood, ×100; (C) mild infiltration of the lymphocyte (circle) in the pars nervosa of the pituitary observed at 14^th^ DPI, H&E, ×400; (D) increased number of the pituicytes (arrowhead) observed in the pars nervosa of the pituitary, 14^th^ DPI, H&E, ×400; (E) at 30^th^ DPI, showing an increased number of pituicytes (curved arrow) with an enlarged nucleus in the pars nervosa, H&E, ×200 and (F) at 60^th^ DPI, infiltration of the eosinophils (circle) between the glandular epithelium of anterior pituitary, H&E, ×400.

On 7^th^ DPI, the vascular plexuses/sinusoids of the adenohypophysis (pars distalis, pars intermedia, pars nervosa) of BTV-infected ewes were engorged with homogenous, light eosinophilic fluid. The acidophil (α) cells in the anterior pituitary were swollen with pyknotic nuclei in comparison to the sham control (Fig. 2A,B). In addition to microhaemorrhages, oedema, focal lymphocytic infiltration and increased cellularity (pituicytes) of the neurohypophysis (posterior pituitary) on the 14^th^ DPI, vascular plexuses were prominent with engorged blood at the interface of the anterior pituitary and neurohypophysis (Fig. 2C,D). The noteworthy histopathological changes were not observed in 30^th^ DPI. On the 45^th^ DPI, however, there were more pituicytes found among the nonmyelinated axonic fibres of the posterior pituitary (Fig. 2E). Further, the adenohypophysis showed mild focal infiltrates of eosinophils in the connective tissue stroma on 60^th^ DPI (Fig. 2F). The histopathological changes in the pituitary gland of control sheep were nonsignificant throughout the experimental period.

#### Thyroid gland

**Figure 3.**
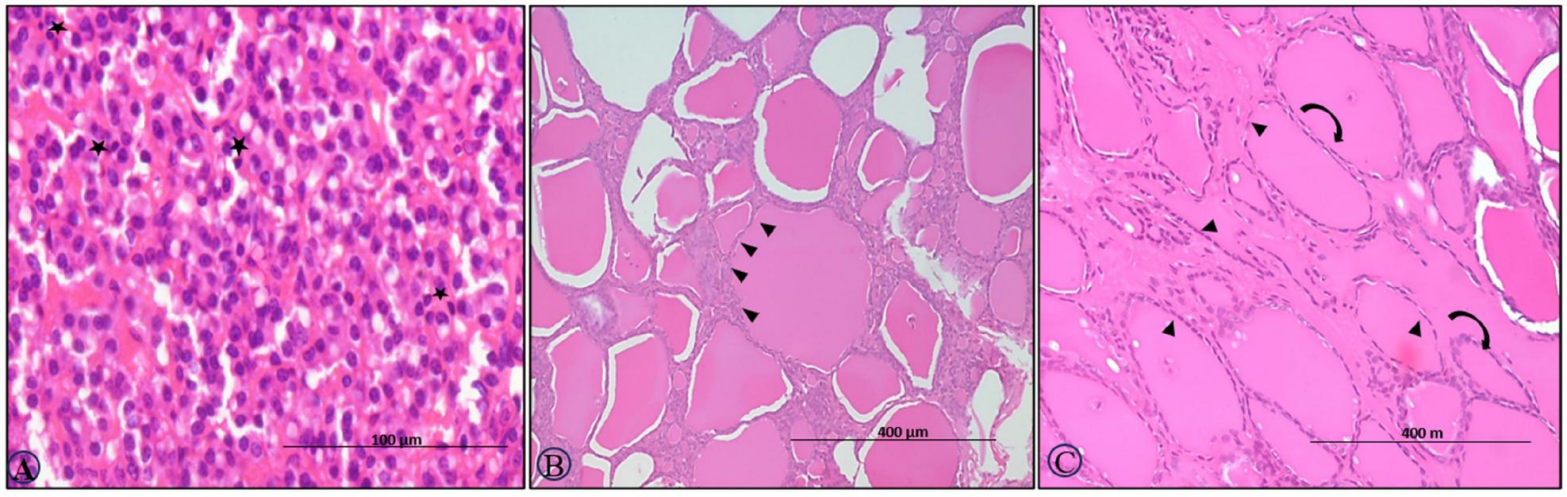
BTV-1, 6 ml (5.5 log10 TCID_50_/ml) inoculated by I/D and IV routes in sheep at 60^th^ gestation day. H&E section, thyroid sections: (A) at 7^th^ DPI, hyperplasia of the parafollicular cells (C-cells) with few undergoing apoptosis (asterisk), ×400; (B) at 30^th^ DPI, enlarged follicles with flattened epithelium (arrowhead) and with dark to lightly stained collide devoid of reabsorption vacuoles, ×200 and (C) at 30^th^ DPI, showing elongated follicle filled (curved arrow) with the colloid with flattened follicular epithelial cells (arrowhead) on 45^th^ DPI, H&E, ×200.

On the 7^th^ DPI, the thyroid glands showed lobules with follicles of variable morphology filled with darkly pink colloid and lined with cuboidal follicular epithelial cells (T-3, T-4 hormones) with resorption vacuoles, especially in the peripheral follicles. Furthermore, multi-focal hyperplasia of C-cells (calcitonin-secreting cells) in the vascular-rich stroma was observed in the walls of adjoining follicles. The parathyroid small cells (parathormone-secreting chief cells) showed distinct vacuolation of the cytoplasm (Fig. 3A).

The follicle morphology on the 14^th^ DPI was comparable to that on the 7^th^ DPI. The C-cells and oxyphilic cells, in contrast, did not exhibit any remarkable alterations. On day 30, the thyroid follicles were mostly enlarged in size, filled with darkly stained colloid with no resorption vacuoles and lined with squamous follicular cells. When compared to controls, numerous small sized follicles were found congregated among larger follicles (Fig. 3B). On days 45 and 60, the thyroid glands remained similar in histopathological details as on 30^th^ DPI (Fig. 3C). The thyroid glands of control ewe were unaffected throughout the experimental period, having normal size follicles filled with colloid and normal tissue stroma.

#### Adrenal gland

**Figure 4.**
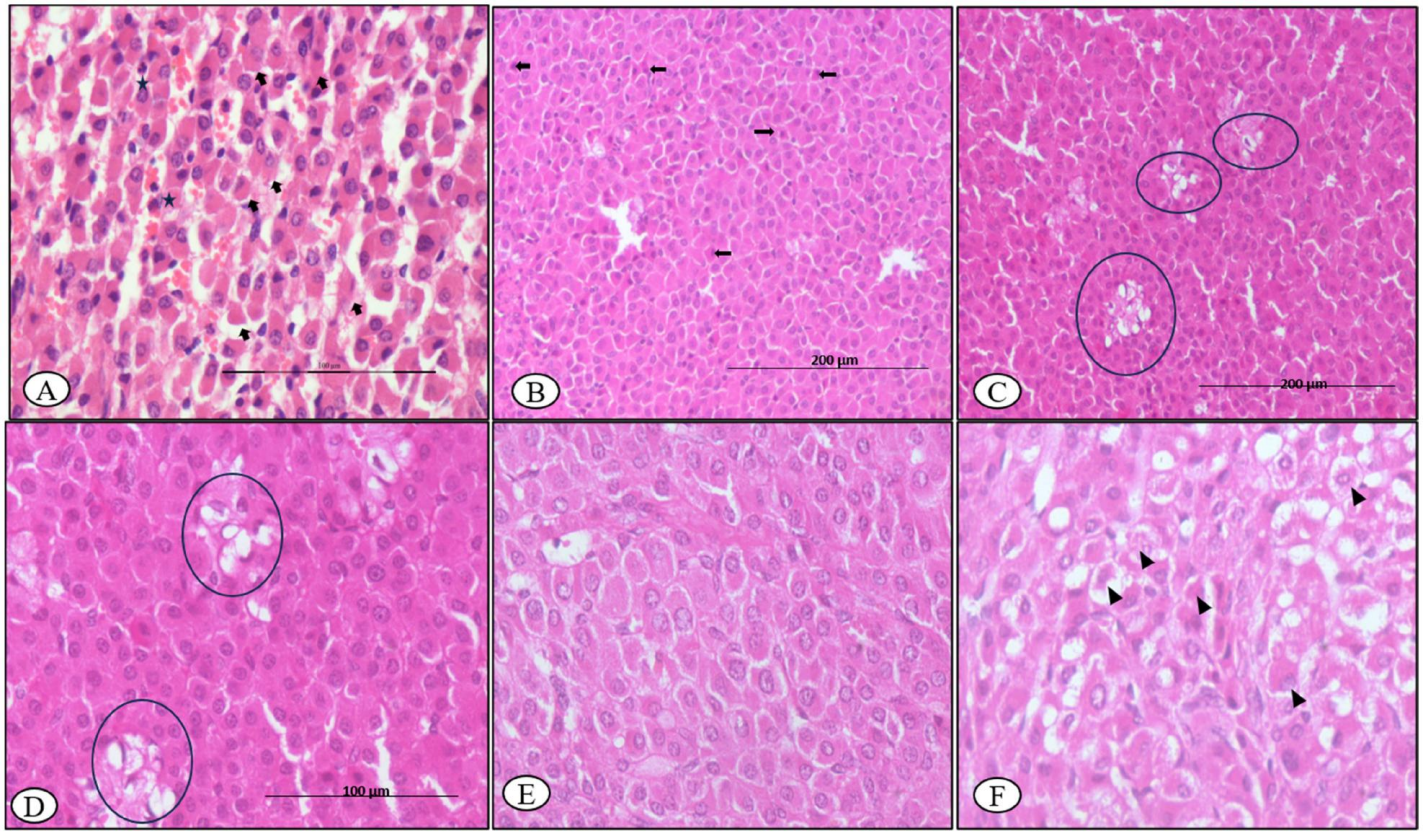
BTV-1, 6 ml (5.5 log10 TCID_50_/ml) inoculated by I/D and IV routes in sheep at 60^th^ gestation day. H&E section, adrenal sections: (A) at 15^th^ DPI, distorted histoarchitecture with dilated sinusoids containing sparse neutrophils (asterisk) and a few degenerated cells in the zona fasciculate (arrow), ×200; (B) at 15^th^ DPI, few cells in the glomerulosa have a condensed nucleus, hyper-eosinophilic cytoplasm and distinct boundaries (arrow), ×200; (C) at 30^th^ DPI, clumps of the ruptured cells vacuolated cells with eccentrical nuclei in zona glomerulosa (circle), ×200; (D) a higher magnification of (C), ×400; (E) at 45^th^ DPI, hypertrophy and hyperplasia, cell of zona glomerulosa, H&E, ×400 and (F) at 60^th^ DPI, section showing a degenerated cell (arrowhead) in the zona fasciculata H&E, ×400.

On 15^th^ DPI, HE sections of adrenals showed distorted histoarchitecture with dilated sinusoids containing a sparse number of neutrophils and a few scattered degenerated endocrine cells secreting cortisol as well as cortisone in the zona fasciculata of the adrenal cortex (Fig. 4A). The cells in the adrenal cortex were multicentrically dropped out from the surrounding cells and were having condensed eosinophilic cytoplasm and nuclei (apoptotic cells; Fig. 4B). At 30^th^ DPI, clumps of the ruptured vacuolated cells with eccentric nuclei were observed in the zona glomerulosa (Fig. 4C,D). Further, hypertrophy and hyperplasia of cells secreting corticosterone and aldosterone of zona glomerulosa were found at the 45^th^ DPI (Fig. 4E). At 60^th^ DPI, adrenal glands showed degenerated cells in the zona fasciculata (Fig. 4F).

**Figure 5.**
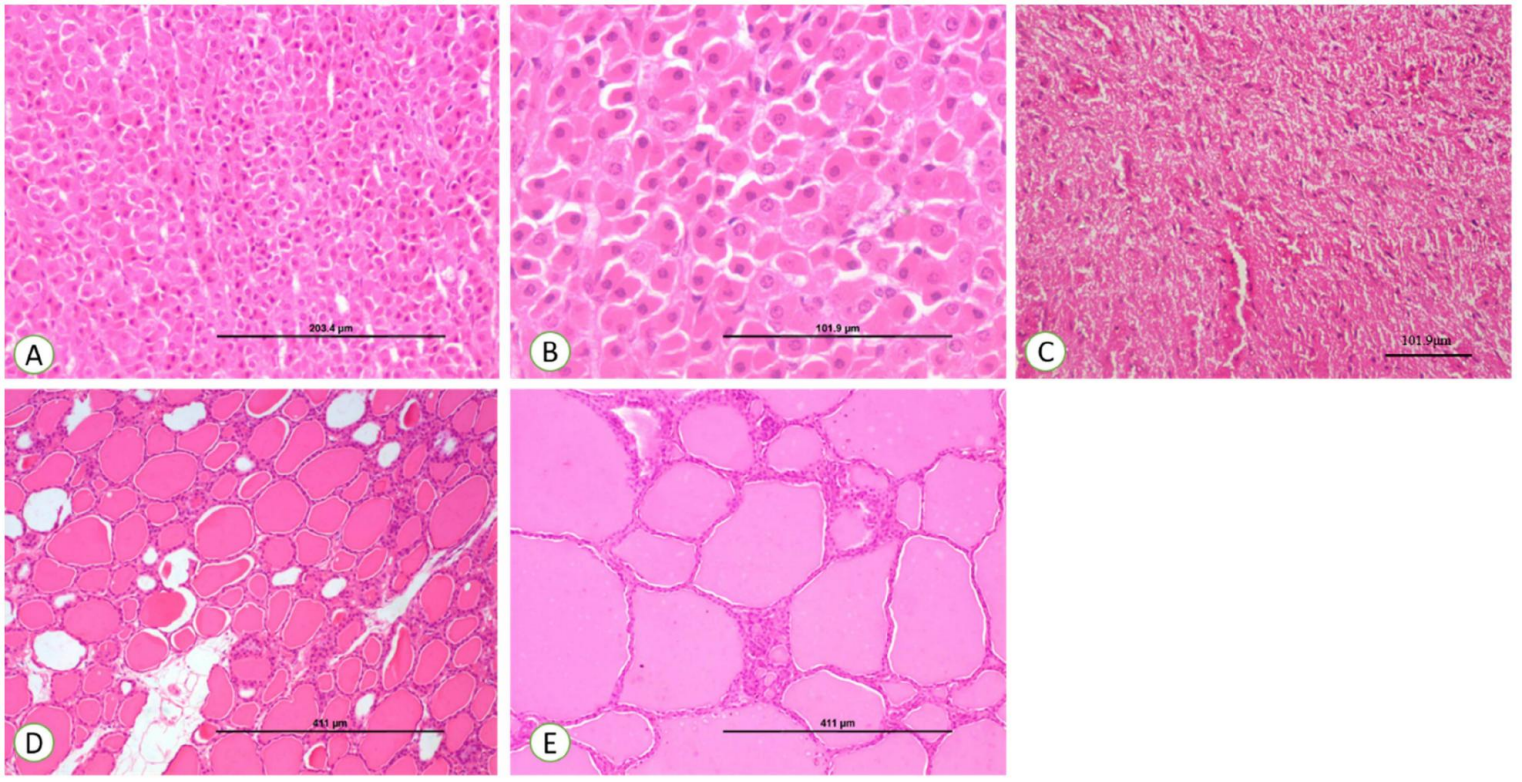
(A) Control section of the adrenal gland, ×200. (B) Control section of the adrenal gland, ×400. (C) Control section of thyroid, ×100. (D) Control section of thyroid, ×200. (E) Control section of the pituitary gland showing normal pituicytes, ×200.

Histology of adrenal, thyroid and posterior pituitary of control ewes showed no significant morphological changes throughout the experimental trial (Fig. 5A–E).

#### Pancreas

**Figure 6.**
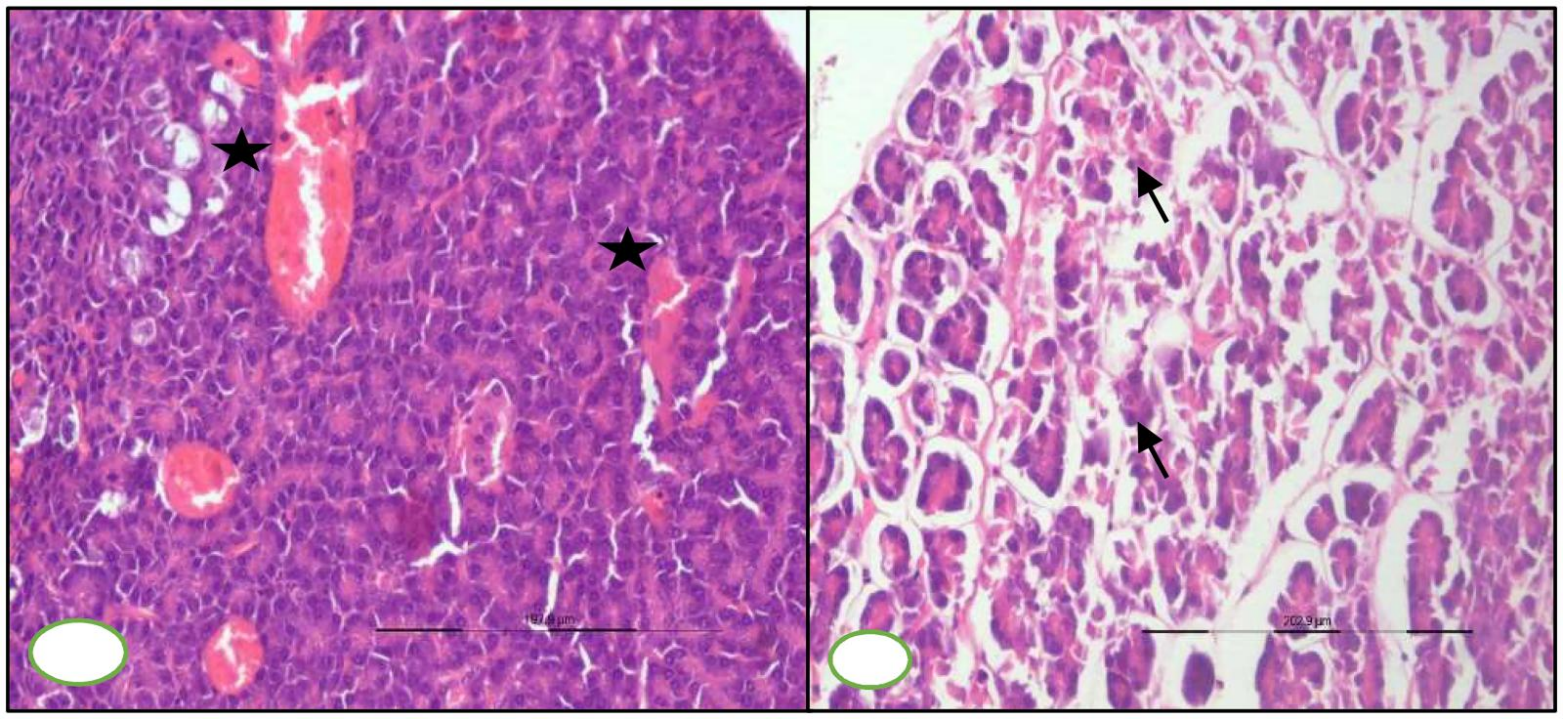
BTV-1, 6 ml (5.5 log10 TCID_50_/ml) inoculated by I/D and IV routes in sheep at 60^th^ gestation day. H&E section, pancreases sections: (A) at 15^th^ DPI, engorged capillaries (asterisk) in the parenchyma, ×200 and (B) at 30^th^ DPI, disorganized atrophic pancreatic lobules characterized degenerated exocrine acini (arrow) having pycnotic nuclei and darkly stained cytoplasm, ×200.

At 7^th^ DPI, congested blood vessels were observed in the pancreas section, microscopically. Pancreatic section revealed atrophy of pancreatic acini and interlobular septa oedema on microscopic examination at 30^th^ DPI (Fig. 6).

### Viral quantification

**Figure 7.**
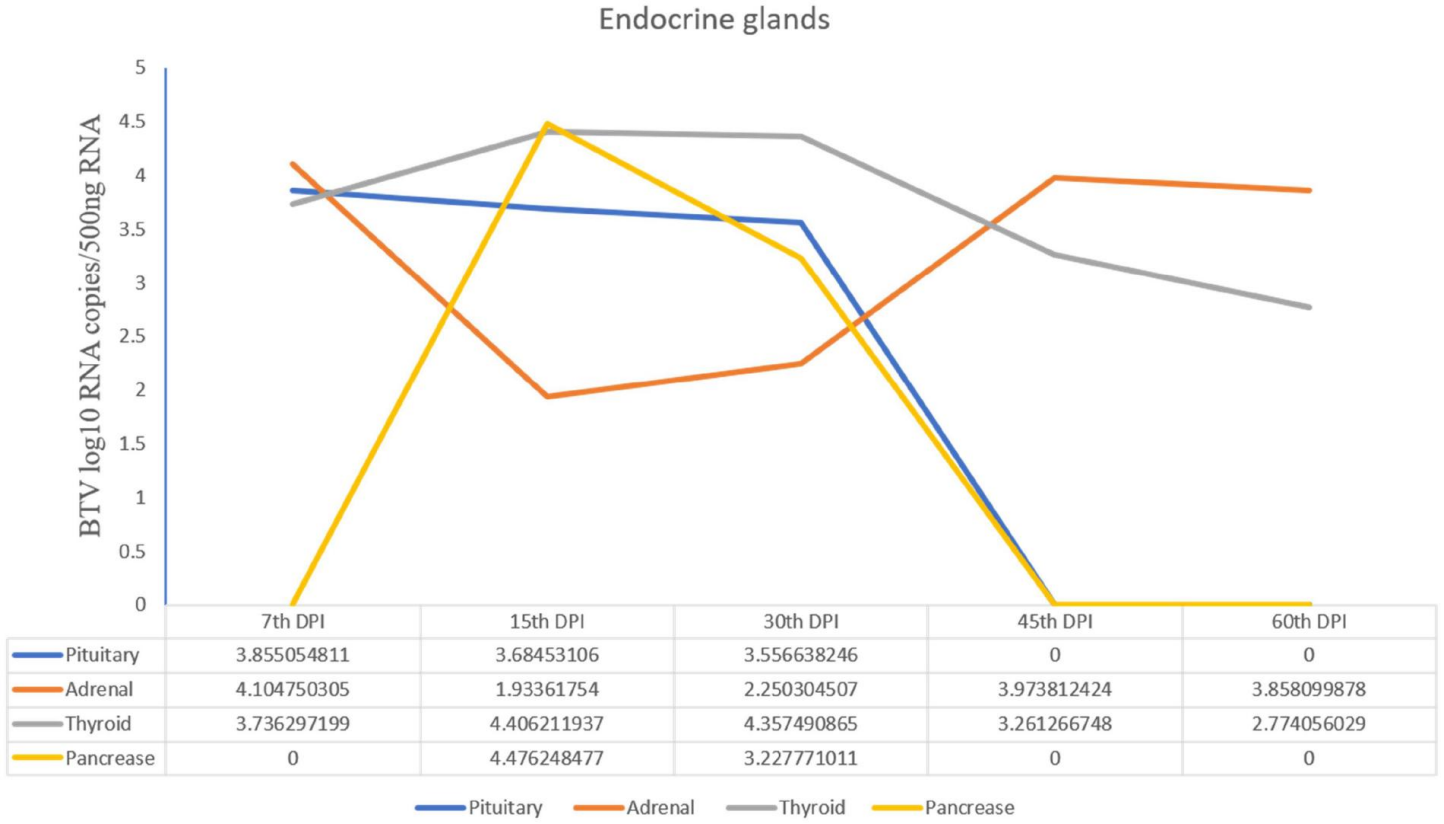
Viral load (Viral copy number of BTV) in the pituitary, adrenal and thyroid glands of infected animals at different time points.

The BTV-1 load in the pituitary gland at 7^th^ DPI and thyroid gland at 15^th^ DPI was highest with the viral copy number (log10 BTV copies/500 ng Tissue RNA) 3.8 and 4.4, respectively. In addition, the adrenals showed the viral load (copy number-4.1) at 7^th^ DPI, which reduced on subsequent days of sacrifice (i.e., 15^th^, 45^th^ and 60^th^ DPI) as compared to the control. The highest viral copy number of BTV in pancreas tissue was observed at 15^th^ DPI (Fig. 7).

### Immunolocalization

**Figure 8.**
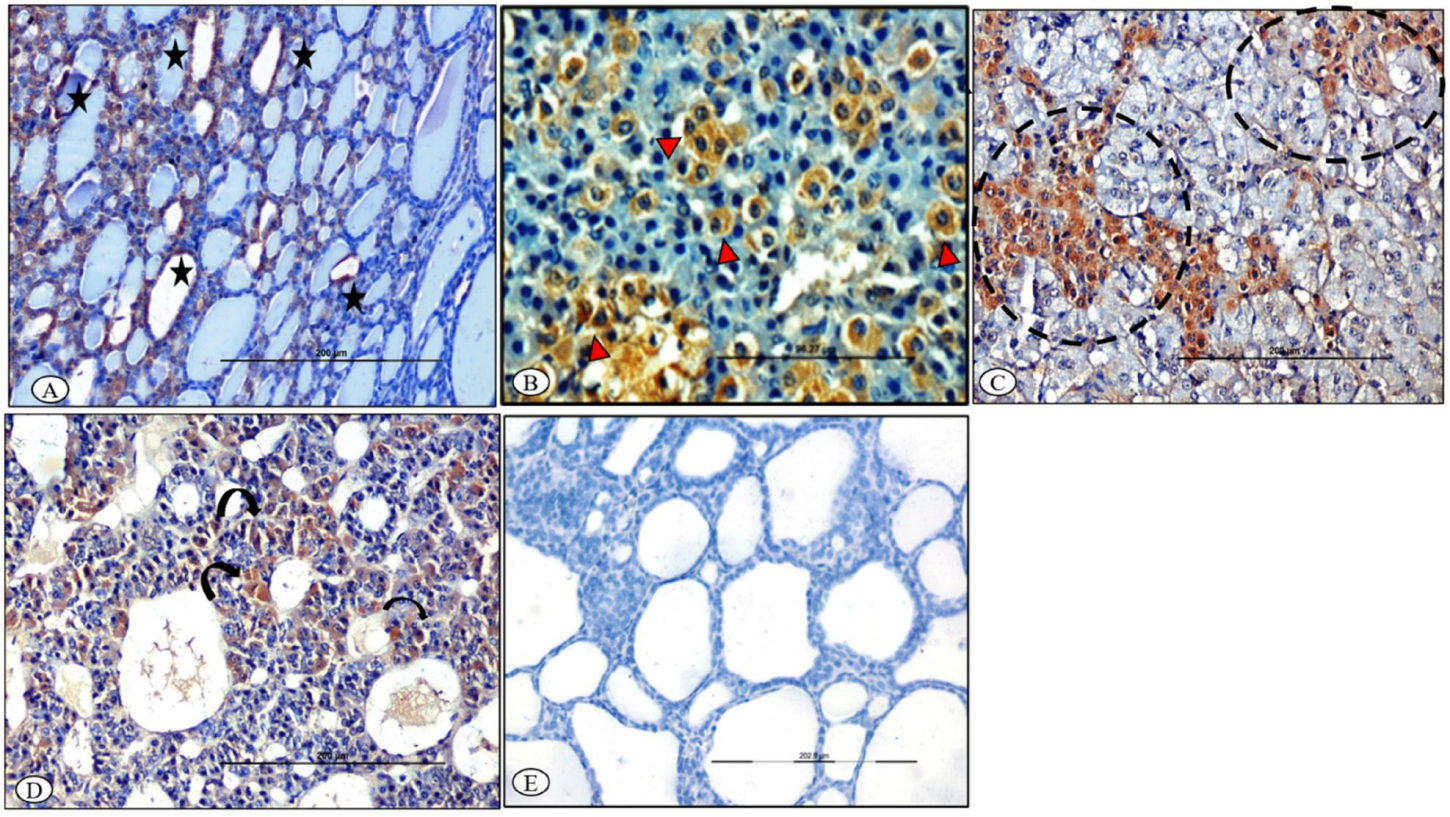
BTV-1 infected sheep at 65^th^ DPI, ABC-IHC-DAB, immunohistochemically stained section, polyclonal BTV-1 primary antibody. (A) Thyroid section showing positive immunolabeling in the cytoplasm of the follicular epithelial cell (asterisk) at 14^th^ DPI, ×200. (B) Adrenal gland section showing positive immunolabelling in the cytoplasm of the cell of zona fasciculata (arrowhead) at 7^th^ DPI, ×400. (C) Adrenal gland section showing positive immunolabelling in the cytoplasm of the cell of zona reticularis projecting in the medullary region (dotted circle) of the gland at 14^th^ DPI, ×400. (D) Pituitary gland showing positive immunolabeling in the cytoplasm of the cell of the adenohypophysis (curved arrow) at 14^th^ DPI, ×200. (E) Antibody control section devoid of any immunolabeling, IHC-DAB, 14^th^ DPI, ×200.

At 7^th^ DPI, BTV-1 antigen was localized in the cytoplasm of the follicular epithelial cells of various lobules and parafollicular cells of the stroma in the thyroid glands of infected ewes (Fig. 8A). In the adrenal glands, BTV-1 antigen was localized in the degenerated cells of zona fasciculata, and in the radiating cells of zona reticularis at 7^th^ DPI (Fig. 8B,C). Further, cells surrounding the sinusoids had variable (mild-to-moderate) degrees of immunolabeling in pituitary adenohypophysis at 7^th^ DPI (Fig. 8D). The control sections of the thyroid glands were devoid of any immunolabeling (Fig. 8E).

### Immunolocalization of caspase-3

**Figure 9.**
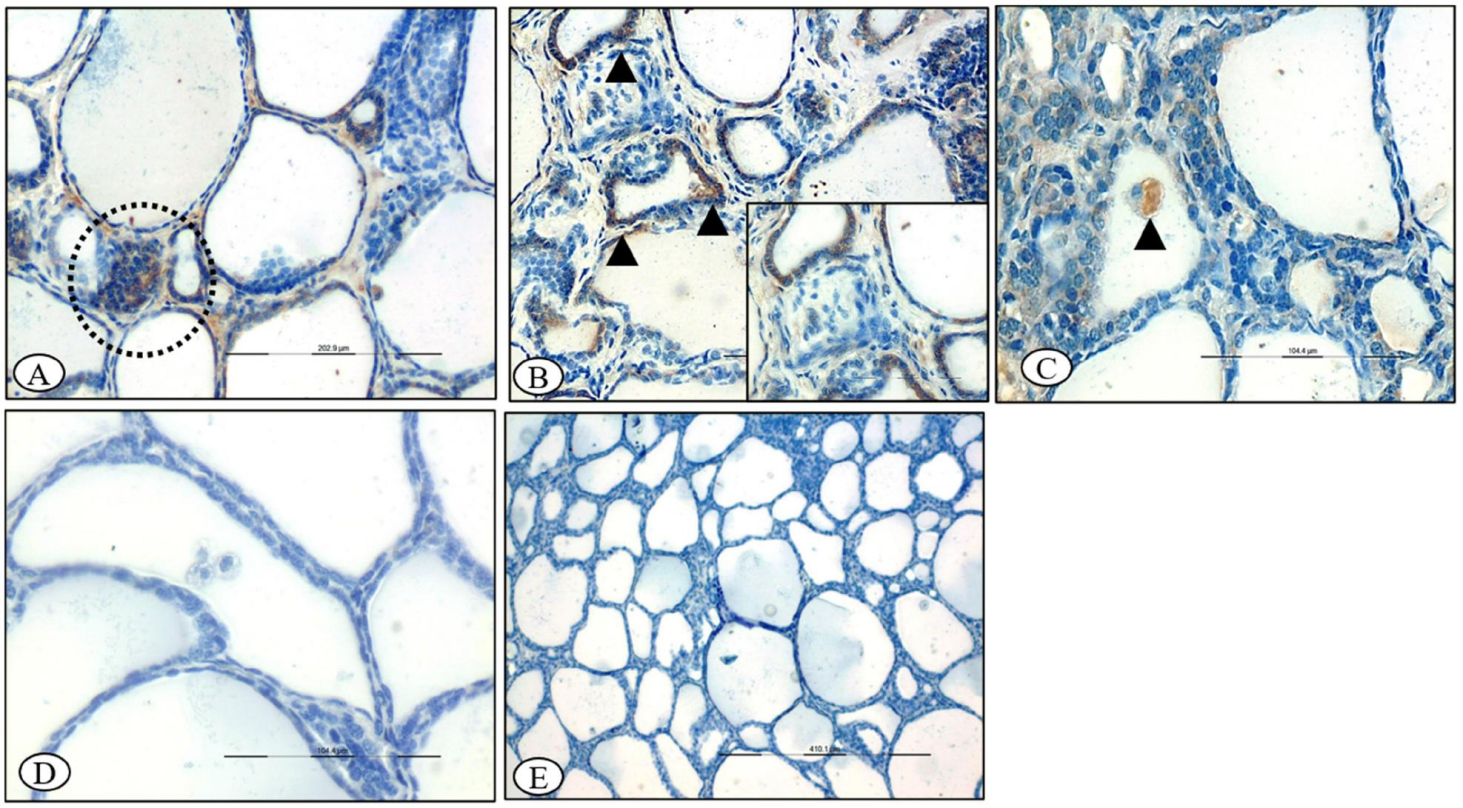
BTV-1 infected sheep at 60^th^ DPI, ABC-IHC-DAB, immunohistochemically stained section, polyclonal caspase-3 primary antibody. (A) Thyroid follicles showing the positive immunostaining for anti caspase-3 in the parafollicular cells of the thyroid as well as in the cytoplasm of the endothelial cell lining the blood vessels (circle). (B) Thyroid follicular epithelial showing the positive immunostaining for anti caspase-3 in the follicular epithelial cells (arrowhead), 30^th^ DPI, DAB-IHC, ×200 (*inset:* higher magnification, ×400). (C) Positive immunolabeling in the cytoplasm of the infiltrated macrophage (arrowhead), 30^th^ DPI, ×400. (D) No signals were detected in the control group thyroid sections, 30^th^ DPI. (E) No immunostaining was observed in the primary antibody control groups, ×400.

**Figure 10.**
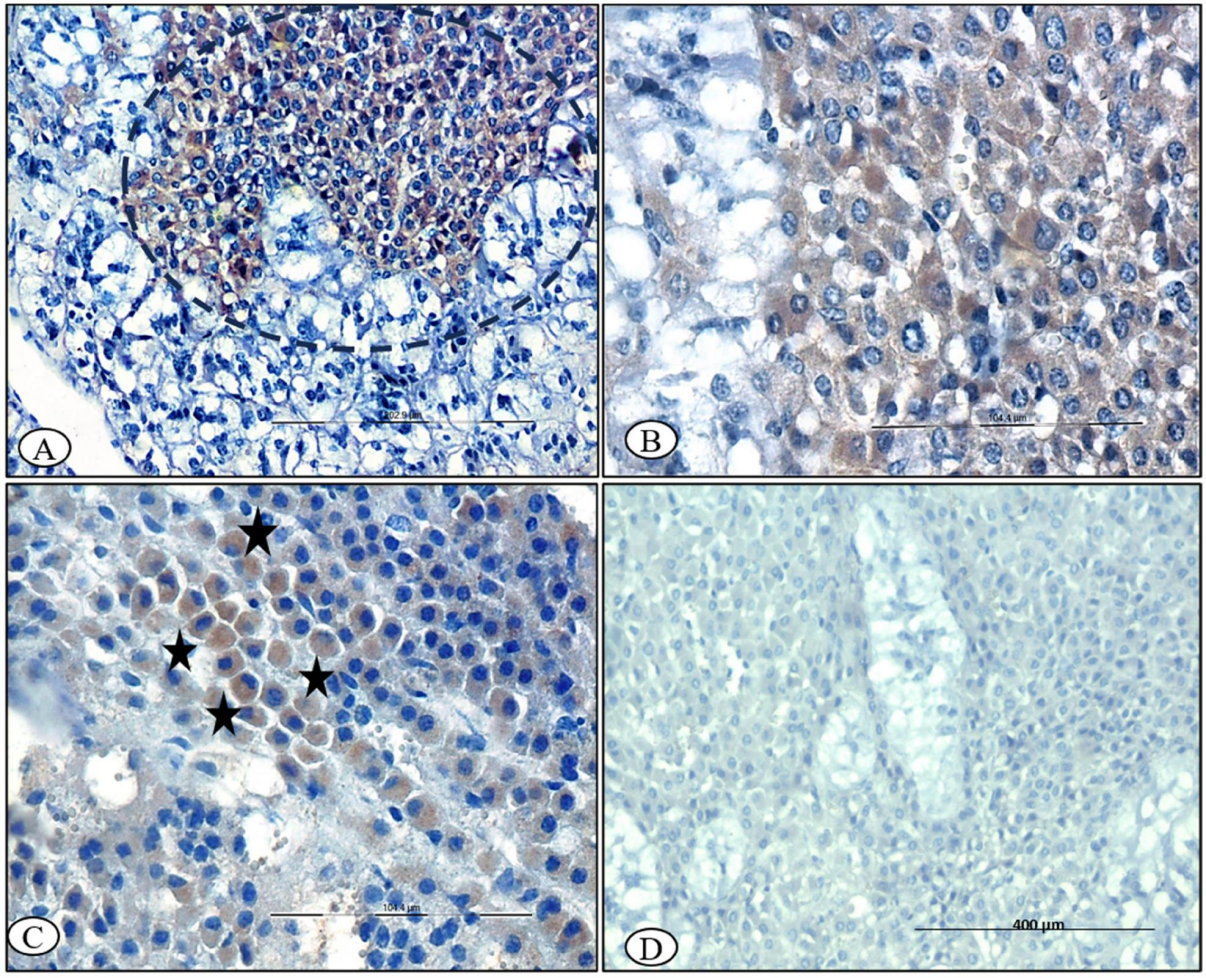
BTV-1 infected sheep at 60^th^ DPI, ABC-IHC-DAB, immunohistochemically stained section, polyclonal caspase-3 primary antibody. (A) Adrenal gland showing the positive immunostaining for anti caspase-3 in the cytoplasm of the cell of zona reticularis cells (circle), ×200. (B) Higher magnification, ×400. (C) Positive immunolabeling in the cytoplasm of zona fasciculata cells (asterisk) that arranged in the linear manner, ×400, 30^th^ DPI. (D) No signals were detected in the control group adrenal sections, 30^th^ DPI, DAB-IHC, ×100.

In the thyroid gland, C-cells occasionally contained mild-to-moderate cytoplasmic immunoreactivity for caspase-3 at 15^th^ DPI (Fig. 9A). Moreover, thyroglobulin synthesizing follicular cells showed mild cytoplasmic immune-reactivity for caspase-3 at 15^th^ and 30^th^ DPI (Fig. 9B,C). The control section was devoid of immunolabeling (Fig. 9D,E). In the adrenal gland, positive immunolabeling in the cells of the zona reticularis adjacent to the chromaffin cells of the medulla was observed at 15^th^ DPI (Fig. 10A). The glucocorticoid-producing cells of the zona fasciculata were mildly stained for caspase-3 at 30^th^ DPI. The cells showed a condensed nuclei and cytoplasm, and cells retracted from the adjacent cells at 30^th^ DPI (Fig. 10B,C). The adrenal medulla chromaffin cells were negative for caspase-3 at 7^th^, 15^th^ and 30^th^ DPI. Control sections were devoid of any immunolabeling at every time point (Fig. 10D).

### Serum hormone concentration

Plasma progesterone, cortisol and thyroid hormone (T-3, T-4), concentrations were measured in the BTV-1 and sham infected (control group) ewes. Mean ± standard error of hormone concentration is presented in Table 1.

**Table 1.** Serum hormone concentration (mean ± SE) in the mid-infected group (GrIMP) and control group at different days postinfections.

	DPI						
	0^th^ DPI	3^rd^ DPI	7^th^ DPI	14^th^ DPI	21^th^ DPI	45^th^ DPI	60^th^ DPI
*Cortisol*							
GrIMP	56.19 ± 19.9*	83.19 ± 16.14	78.85 ± 21.87	64.62 ± 23.82	78.39 ± 18.02	74.19 ± 43.08	54.66 ± 54.85
Control	140.57 ± 17.1	87.12 ± 17.13	100.35 ± 10.62	78.58 ± 5.06	77.92 ± 7.69	78.58 ± 3.58	78.58 ± 5.06
*T-3*							
GrIMP	6.9 ± 3.72*	9.34 ± 2.1*	8.43 ± 3.07	10.2 ± 11	13.5 ± 10.67	11.05 ± 2.76	7.37 ± 8.53
Control	23.06 ± 4.18	22.74 ± 4.04	7.31 ± 1.3	9.32 ± 0.16	7.49 ± 0.81	9.13 ± 0.15	9.49 ± 1.32
*Thyroxine (T-4)*							
GrIMP	64.69 ± 32.24	61.04 ± 33.12	50.51 ± 33.55	50.23 ± 33.17	73.29 ± 32.62	107.46 ± 49.81	125.97 ± 50.94
Control	31.37 ± 8.98	24.07 ± 10.62	28.64 ± 6.62	26.11 ± 11.93	27.55 ± 4.68	25.99 ± 5.18	32.39 ± 7.33
*Progesterone*							
GrIMP	27.01 ± 10.41*	28.17 ± 9.25*	37.97 ± 13.14	43.24 ± 10.54	27.33 ± 8.86*	43.65 ± 24.73	43.65 ± 24.73
Control	77.2 ± 3.77	74.73 ± 3.6	39.21 ± 14.68	42.17 ± 18.48	59.12 ± 9.5	50.99 ± 7.4	23.5 ± 2.74

Data are presented in the form of mean ± SEM. Statistical analysis was performed using paired *T*-test. *P values less than 0.05 were considered statistically significant.

### Kinetic studies of progesterone (P4)

In BTV-1 infected groups, the upsurge in P4 concentration level (43.23 nmol/l) up to 14^th^ DPI, then abruptly drop (27.32 nmol/l) by 21^st^ DPI and later on-again upsurge was observed in subsequent DPI. A similar trend in the mean value of the P4 concentration was observed in the mock infected control group ewes.

### Kinetic studies of triiodothyronine (T-3)

BTV-1 infected ewes showed an increasing trend in mean T-3 concentration from 0^th^ (6.8 nmol/l) to 90^th^ (37.14 nmol/l) DPI, when compared with the preinoculated stage. In contrast, the control group ewes showed a decreasing trend in mean T-3 concentration from 0^th^ (23.05 nmol/l) to 90^th^ (7.38 nmol/l) DPI.

### Kinetic studies of thyroxine (T-4)

The BTV-1 infected ewes showed the reduction in mean T-4 concentration from 0^th^ (64.69 nmol/l) to 14^th^ (50.23 nmol/l) DPI, which further surge to 90^th^ DPI in comparison to the preinoculation stage.

### Kinetic studies of cortisol

The slight increment in mean cortisol serum level from 7^th^ (56.18 g/dl) to 15^th^ DPI (78.84 g/dl) was observed in BTV-1 infected ewes compared to the preinfected stage. However, cortisol upsurging was not statistically significant when compared with the control ewes at equivalent days. The cortisol level in the BTV-1 infected ewes in late phase of infection and control ewes was similar.

## Discussion

BT is an endemic and reemerging disease of ruminants, particularly of sheep and other wild ruminants, transmitted by blood-sucking midges. BT control is a major challenging task because of the existence of multiple BTV serotypes, and cropping up of new serotypes from time to time in different geographical regions. The pathogenic potential of these serotypes has not been completely delineated. The tropism of BTV for vascular endothelial cell consequences pathology in the organ where vascular endothelial cells are prominent. The virus-induced inflammatory lesions like coronitis, striated muscle lesions, skin lesions, foetal pathology-cavitating lesions in the brain, abortions/stillbirths and wool defects have been documented. However, the pathogenic potential of BTV serotypes in the secretory epithelial cell of ductless endocrine glands such as pituitary (ectoderm origin), thyroid (endoderm origin) and adrenal glands (intermediate mesoderm and neuro-ectoderm origin) has not been portrayed extensively. Therefore, the BTV-1 serotype-induced endocrine pathology has been portrayed in this study, which is often ignored during the natural outbreaks of BT disease. Herein out of naturally mated 15 pregnant ewes, 10 ewes were experimentally infected with BTV-1 serotype, while the remaining 5 ewes were mock infected with cell culture fluid at the 60^th^ day of their gestation. Both treatment groups were monitored and sacrificed at different time points for the collection of pituitary, thyroid and adrenal glands.

Pituitary gland regulates most of the target endocrines such as thyroid and adrenals. In the BTV-1 infected ewes, pituitaries showed oedematous changes and engorged capillary plexuses/sinusoids grossly as well as microscopically, which might be due to the higher proinflammatory cytokine levels and enhanced permeability as the proinflammatory cytokines and vasoactive mediators accounted for oedematous and haemorrhagic changes in BTV experimentally infected animals.^25^ Moreover, the microscopically observed congestion in the adenohypophysis and neurohypophysis might be due to enhanced vascular permeability or vascular leakage. The positive immunolocalization of the BTV antigen in secretory epithelial cells of the anterior pituitary and detection of viral RNA in the pituitary glands clearly pointed towards virus mediated pathological consequences. Microscopically, pituicytes hyperplasia might be an adaptive response of the virus-infected gland, which needs further elucidation. The BTV-1 antigen was primarily localized in the cytoplasm of glandular epithelial cells in comparison to other pituitary anatomical regions (intermediate or posterior pituitary cells), indicating a preference of BTV-1 for the anterior pituitary. Thus, the present work ruled out the pituitary cells as a novel site for virus tropism.

The BT viral RNA was detected in adrenal tissue of BTV-1 infected ewes, which is consistent with earlier research.^26^^,^^27^ BTV antigen was localized by the IHC to rule out the possibility that the detection was attributable to the RBC-related virus or infected adrenal cells.

The robust DAB signals in the cells of the zona glomerulosa and the zona fasciculata at 7^th^ and 15^th^ DPI, indicating that BTV-1 infects the cells of the adrenal gland cortex. This observation may be due to the progenitor cells being retained, which makes it more receptive to the BTV as the adrenocortical progenitor cells of the subcapsular cortex, which descend and migrate into the adrenal medulla, maintain cortical homeostasis.^28^ However, the further detail study is warranted to describe the preferentially tropism of BTV towards progenitor lineage.

BTV-related loss of progenitor neuronal and/or glial cells of the central nervous system has been documented previously.^29^ The positive caspase-3 signals in the zona reticularis of the adrenal cortex depicted the apoptosis in infected cells. However, the BTV antigen could only be localized in the cortical secretory epithelial cells, not in the endothelial cells. Together, the absence of immunolabeling in arterioles and capillary endothelial cells and positive immunolabeling from zona glomerulosa and zona reticularis at 7^th^ and 15^th^ DPI pointed towards viral-induced and cytokines-mediated direct injury. The effect of BTV-1 on thyroid gland was portrayed via nucleic acid detection and antigen localization in the follicular epithelial cells of the thyroid gland. Vascular alterations in the thyroid gland, such as blood capillary enlargement and endothelial damage, were in line with the previous findings.^30^ BTV tropism towards the follicular epithelial cells was further confirmed by positive immunolabeling of follicular epithelial cells for BTV antigen. Although morphological indications of apoptosis viz. apoptotic bodies in the flattened follicular epithelial cells were not well evident in the HE sections, however, mild-to-moderate immunosignals against caspase-3 in the cytoplasm of the follicular cells bestowed apoptotic alterations in the thyroid follicular cells. Another possibility for such pathological consequences is that BTV-1 uses the lytic route to exit the follicular epithelial cells (i.e., BTV-1 caused the destruction of the follicular cells). Both apoptosis and proinflammatory cytokines have been proposed as the major events in the lytic release of BTV from mammalian cells.^31^

BTV-induced apoptosis in bovine and ovine endothelial cells, monocytes and T cells.^32–34^ VP2 and VP5, the viral outer capsid layer components, cause apoptosis in infected cells by activating NF kappa-β and executioner caspase-3.^35^ Moreover, apoptosis in mammalian cells needed, simply, the uncoating of the BTV rather than viral multiplication.^35^

In the BTV-1 infected ewes showed an increased level of the serum T-4 hormone as compared with the control animals. These changes might be due to the reduced conversion of T-4 into T-3. Further, the affected C-cells of the thyroid gland in the infected group may disturb the Ca++ metabolism.

In the later stages of the illness, macrophages frequently act as a professional phagocyte in the thyroid follicular tissue to eliminate apoptotic cells.^36^ In the current investigation, macrophages were seen in some thyroid follicles but not all of them. The heterogeneity in the lineage of the apoptotic cells responsible for macrophage activation might be the cause of this disparity. Earlier studies have shown that pentraxins and collectins are associated with late apoptotic cell uptake, whereas expression of CD43 is involved in an early stage of apoptotic uptake.^37^^,^^38^ It is worth noting that big, fused, varied-sized thyroid follicles emerge in the later stages of the infection, which may be attributable to the clearing of the epithelial cells and basement membrane between the follicles.

There are certain restrictions on the current investigation. Therefore, a greater number of animals in each group must be included in order to confirm the current study’s findings. Furthermore, it is crucial to check the endocrine glands for the virus during naturally occurring BTV epidemics.

It is crucial to expand the experiment to include nonpregnant animals of all ages to clarify the virus’s effect on endocrine glands. This broader perspective will enable a more thorough comprehension of the virus’s impacts on the endocrine system.

## Conclusion

The tropism of the BTV-1/SKN-10 for the secretory epithelial cells of the pituitary, thyroid and adrenal glands has been explored in this study. Immunohistochemical localization of the BTV antigen in the glandular epithelial cells of the pituitary, thyroid and adrenal glands of infected ewes has been shown for the first time, which evidently signifies the role of BTV-1 in the pathology of the endocrine glands. Consequently, growth and production will be affected critically. Based on the present findings, further experimental studies are warranted to underpin a molecular basis of the sequential pathological changes related to BTV infection. Moreover, the present findings will pave the way in better understanding of the pathogenesis in a primary host, which is prime prerequisite for disease control.
